# Analyzing single cell transcriptome data from severe COVID-19 patients

**DOI:** 10.1016/j.xpro.2022.101379

**Published:** 2022-04-21

**Authors:** Nasna Nassir, Richa Tambi, Asma Bankapur, Noushad Karuvantevida, Hamdah Hassan Khansaheb, Binte Zehra, Ghausia Begum, Reem Abdel Hameid, Awab Ahmed, Zulfa Deesi, Abdulmajeed Alkhajeh, K.M.Furkan Uddin, Hosneara Akter, Seyed Ali Safizadeh Shabestari, Mellissa Gaudet, Mahmood Yaseen Hachim, Alawi Alsheikh-Ali, Bakhrom K. Berdiev, Saba Al Heialy, Mohammed Uddin

**Affiliations:** 1 College of Medicine, Mohammed Bin Rashid University of Medicine and Health Sciences, Dubai, UAE; 2 Dubai Health Authority, Microbiology and Infection Control Unit, Pathology and Genetics Department, Latifa Women and Children Hospital, Dubai, UAE; 3 Medical Education & Research Department, Dubai Health Authority, Dubai, UAE; 4 Genetics and Genomic Medicine Centre, NeuroGen Children’s Healthcare, Dhaka, Bangladesh; 5 Meakins-Christie Laboratories, Research Institute of the McGill University Health Center, Montreal, QC, Canada; 6 Dubai Health Authority, Dubai, UAE; 7 Cellular Intelligence (Ci) Lab, GenomeArc Inc., Toronto, ON, Canada; 8 Department of Biotechnology, Bharathidsasan University, Tiruchirappalli 620024, Tamilnadu, India

**Keywords:** Bioinformatics, Health Sciences, Genomics, RNAseq, Immunology, Molecular Biology, Gene Expression

## Abstract

We describe the protocol for identifying COVID-19 severity specific cell types and their regulatory marker genes using single-cell transcriptomics data. We construct COVID-19 comorbid disease-associated gene list using multiple databases and literature resources. Next, we identify specific cell type where comorbid genes are upregulated. We further characterize the identified cell type using gene enrichment analysis. We detect upregulation of marker gene restricted to severe COVID-19 cell type and validate our findings using *in silico*, *in vivo*, and *in vitro* cellular models.

For complete details on the use and execution of this protocol, please refer to Nassir et al. (2021b).

## Highlights

• Step-by-step protocol for single cell transcriptome data clustering and analysis
• Customized cell type marker identification pipeline
• Identification of clusters with enrichment of COVID-19 comorbidity associated genes
• Gene expression studies from nasopharyngeal swabs and spike protein simulated cells

## Graphical abstract

**Figure:**
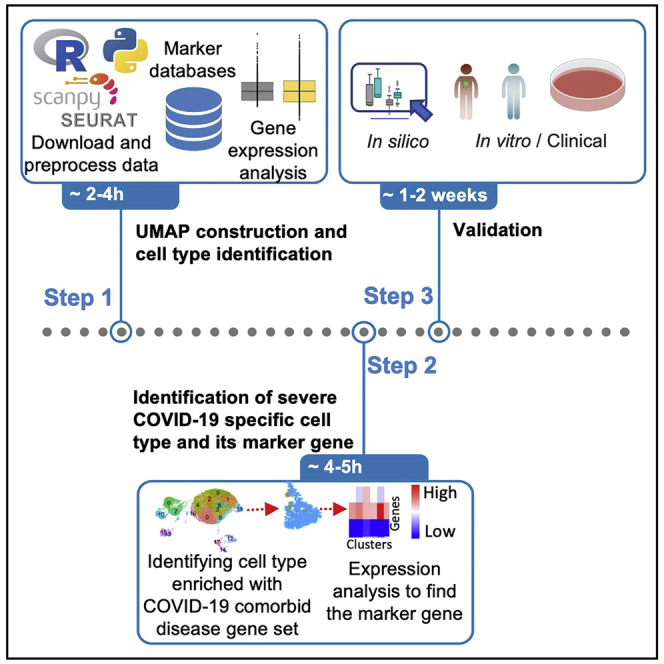

## Before you begin

### Overview

This manuscript details steps in identifying cell types with enriched COVID-19 comorbid genes, which can be used as a severity marker. Comprehensive steps for single-cell RNA-seq data clustering and identification of cell types are discussed. Pathway analysis of the differentially expressed genes of COVID-19 severity specific cell types and detecting their regulatory marker genes are described. Validation studies using *in vitro* (real time PCR for marker gene in nasopharyngeal swabs of COVID-19 patients), *in vivo* (real time PCR in NHBE cells treated with spike protein) and *in silico* (single cell and bulk RNA-seq data) are detailed.

1. Download the relevant datasets.

Download the datasets mentioned in ‘Deposited data’ section of key resources table.

Complete datasets used for this analysis are deposited in Zenodo data: https://doi.org/10.5281/zenodo.5809990.

2. Sample preparation for qPCR from clinical samples.
  a. Collect samples from nasopharyngeal swabs of COVID-19 patients and control group in 3 mL sterile viral transport medium (VTM) tube, following standard sample collection protocol. To maximize the viability of samples, transport all the samples at 2°C–8°C within 72 h post collection and store at −80°C until use.
    **CRITICAL:** Institutional permission and approval to handle SARS-CoV-2 should be taken and any material that has been in contact with the virus should be handled and discarded off in accordance with local regulations.
  b. Keep the following ready.
    i. RNA samples from SARS-CoV-2 infected patients and control group.
    ii. cDNA preparation from samples.
    iii. qPCR probes and kits.
3. Cell culture.
  a. Normal human primary bronchial epithelial (NHBE) cells from non-obese (n=3, 2 male, 1 female) and obese (n=2, male) subjects.
  b. PBMCs from healthy donor (n=1 female).
  c. Culture media and SARS-CoV-2 spike protein.

## Key resources table

**Table:** 

REAGENT or RESOURCE	SOURCE	IDENTIFIER
**Bacterial and virus strains**		

SARS-CoV-2 spike protein (S1+S2)	Sino Biological Inc	Cat# 40591-V08H, 40590-V08B

**Biological samples**		

Nasopharyngeal swab COVID -19 severe patients and control ∗Age-sex details in Table S5 (Nassir et al., 2021b)	Dubai Health Authority	DSREC-04/2020_02

**Chemicals, peptides, and recombinant proteins**		

RiboZol RNA extraction reagent	VWR	Cat# DFU-N580

**Critical commercial assays**		

QIAamp Viral RNA Mini or the EZ1 DSP Virus Kits	QIAGEN	Cat# 955134
RNeasy 96 QIAcube HT Kit	QIAGEN	Cat# 74171
High-Capacity cDNA Kit	Applied Biosystems	Cat# 4368814
Taqman Fast advanced master mix	Thermo Fisher Scientific	Cat# 4444557
AccuRT Genomic DNA Removal Kit	Applied Biological Materials	Cat# G488
All-In-One Reverse Transcriptase Mastermix	Applied Biological Materials	Cat# G592
EvaGreen qPCR Mastermix	Applied Biological Materials	Cat# Mastermix-S

**Deposited data**		

Healthy control airway scRNA-seq	(Deprez et al., 2020)	https://www.genomique.eu/cellbrowser/HCA/HCA_airway_epithelium/exprMatrix.tsv.gz
Healthy control lung scRNA-seq	(Madissoon et al., 2019)	https://cellgeni.cog.sanger.ac.uk/tissue-stability/lung.cellxgene.h5ad
COVID-19 Patient BALF scRNA-seq	(Liao et al., 2020)	GEO: GSE145926
COVID-19 Patient bulk PBMC RNA-seq	(Arunachalam et al., 2020)	GEO: GSE152418
COVID-19 Patient bulk nasopharyngeal RNA-seq	(Lieberman et al., 2020)	GEO: GSE152075
COVID-19 Patient BALF scRNA-seq	(Grant et al., 2021)	GEO: GSE155249
Complete datasets used for this analysis	Zenodo	Zenodo data: https://doi.org/10.5281/zenodo.5809990.

**Experimental models: Cell lines**		

Normal human primary bronchial epithelial (NHBE) cells from non-obese and obese subjects ∗Age-sex details in Table S6 (Nassir et al., 2021b)	MatTek and ATCC or obtained from the Biobank of the Quebec Respiratory Health Research Network at the Meakins-Christie Laboratories, Research Institute of the McGill University Health Centre (GLEN site)	NA

**Oligonucleotides**		

FCGR3B qPCR probe	Applied Biosystems	Hs04334165_m1
FFAR2 qPCR probe	Applied Biosystems	Hs00271142_s1
glyceraldehyde-3-phosphate dehydrogenase qPCR probe	Applied Biosystems	Hs02786624_g1
FCGR3B Forward primer	Thermo Fisher Scientific	GGAGAGTACAGGTGCCAGACAA
FCGR3B Reverse primer	Thermo Fisher Scientific	CCTCAGGTGAATAGGGTCTTCC
GAPDH Forward primer	Thermo Fisher Scientific	GAAGGTGAAGGTCGGAGT
GAPDH Reverse primer	Thermo Fisher Scientific	GAAGATGGTGATGGGATTTC

**Software and algorithms**		

Scanpy (version:1.7.0)	(Wolf et al., 2018)	https://scanpy.readthedocs.io/en/stable/
Seurat (version:3.2)	(Stuart et al., 2019)	https://satijalab.org/seurat/
Custom Source Code	GitHub Folder: MBRULab/2021_Nassir-etal	Github data: https://github.com/MBRULab/2021_Nassir-etal

**Other**		

PanglaoDB	(Franzen et al., 2019)	https://panglaodb.se/
CellMarker	(Zhang et al., 2019)	http://biocc.hrbmu.edu.cn/CellMarker/
Immunology Database and Analysis Portal	(Bhattacharya et al., 2018)	https://www.immport.org/
Cytoscape	(Shannon et al., 2003)	https://cytoscape.org/
Sfari	(Abrahams et al., 2013)	https://gene.sfari.org/
GWAS catalogue	(Buniello et al., 2019)	https://www.ebi.ac.uk/gwas/
NanoDrop™ 8000 Spectrophotometer	Thermo Fisher Scientific, USA	S/N 3274
QuantStudio5™ Real-Time PCR System	Applied Biosystems, USA	S/N 272523107
Thermo mixer with a 96-well PCR plate holder	Eppendorf AG 22331, Hamburg, Germany	S/N 53821O131651
AC-T DIFF cell counter	Beckman Coulter	S/N 6605477
Thermal cycler	Bio-Rad, Hercules, USA	CFX96
QIACube HT Robotic Workstation	QIAGEN, USA	S/N 012648

## Materials and equipment

### Bioinformatics analysis

All the bioinformatics analyses were carried out either on the institute (MBRU) server (Linux OS, AMD EPYC 7402 24-Core Processor, 132 GB RAM) or PC (iMac, 4.3 GHz Intel Core i7 processor, 16 GB RAM). The minimum configuration needed for carrying out this analysis is Linux OS, 16 GB RAM and around 30 GB of available storage space.

## Step-by-step method details

**Timing: ∼2–4 h(Processing the single-cell RNA-seq data)**

**Timing: ∼1 week (data collection), 10 min (script execution)(Prepare signature gene marker dataset and ascertain cell type identity of clusters)**

**Timing: ∼1 week (compiling the comorbid gene list), 15–20 min (executing the script)(Prepare comorbid gene list and identify subtype with upregulated comorbid gene expression)**

**Timing: ∼ 2–4 h(Downstream analysis)**

**Timing: ∼ 1–2 h(Pathway enrichment)**

**Timing: ∼ 1–2 h(In silico validation)**

**Timing: ∼ 1–2 weeks(Gene expression in severe COVID-19 clinical samples)**

**Timing: ∼ 1 week (Lung epithelial cell culture: ∼7 days, Monocyte isolationandculture: 24 h, qPCR: 4 h)(Gene expression in spike protein stimulated obese subjects)**

**Figure 1:**
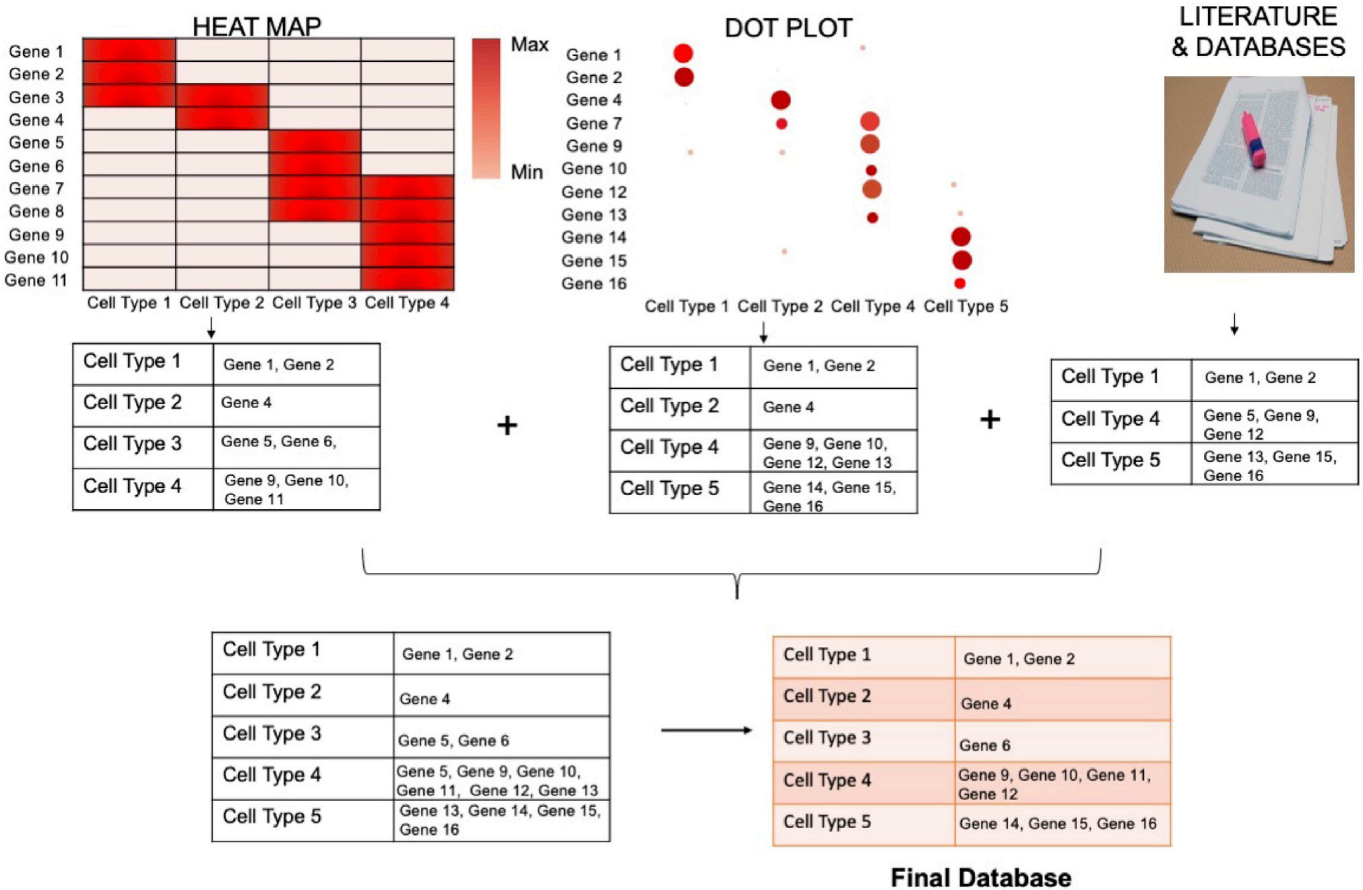
Steps involved in the compilation of unique marker gene database using various sources Marker gene information is present in literature in the form of heat map (Gene expression plotted against cell types using color gradients for expression levels), dot plot (circles denoting the gene expression across various cell types), tables or databases. In case of dot plot, the size and color intensity are proportional to the level of expression in the percent of cells and degree of expression, respectively (in ascending order). In order to constitute the final marker database, the cell types and gene list were combined from all the sources retaining only unique genes into the list for each cell type (Removing any duplicate genes within inter and intra cell types).

**Figure 2:**
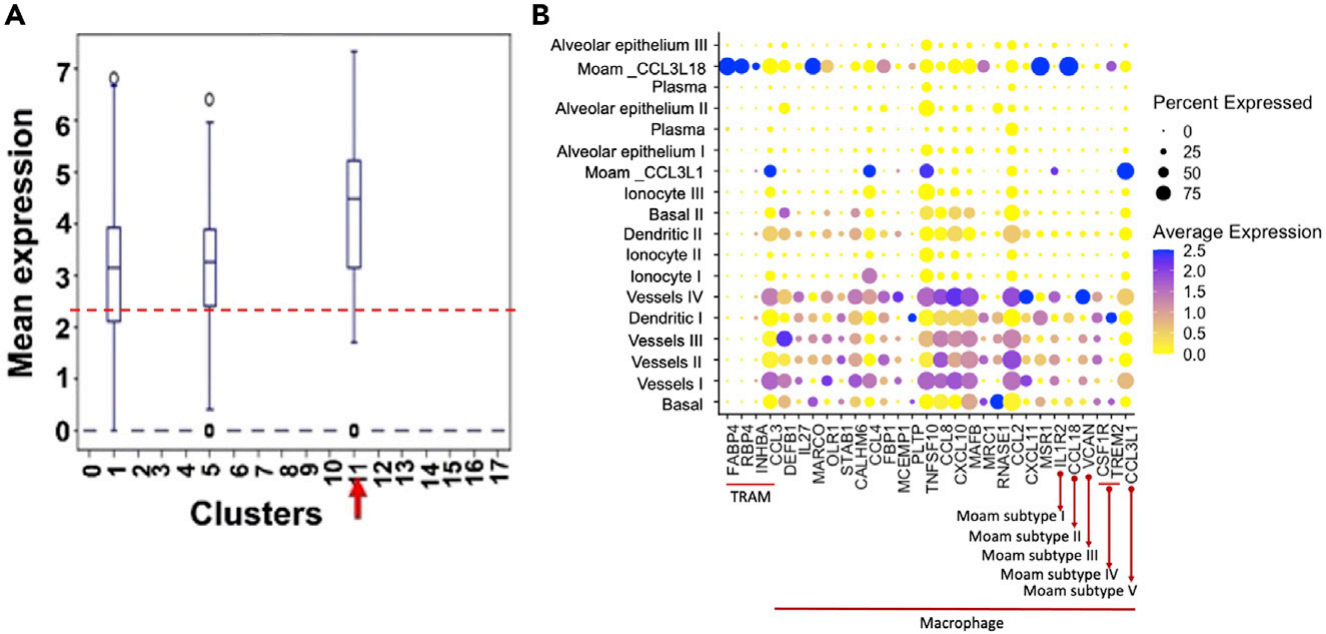
Assigning cell type identity for severe COVID-19 cluster 11 (A) Mean expression of *CCL3L1* across all clusters in severe COVID-19 dataset. The highest expression was observed for cluster 11, which was annotated as monocyte derived alveolar macrophages (MoAM), marked by *CCL3L1*. Red dotted line indicates the global median expression. A cluster was assigned a particular cell type if it had the highest median expression (across the clusters and expression value was more than 99th percentile overall expression. (B) Dot plot showing expression of macrophage and its subtype (TRAM and MoAM) marker genes for the severe COVID-19 dataset. The y-axis represents the cell types based on the marker database and x-axis represents the marker genes. Cluster 11 is marked by MoAM_CCL3L1.

1. Processing the single-cell RNA-seq data.
  This section describes processing of single cell RNA-seq data including the installation of required packages and codes required to execute each step.
  a. Control dataset processing using Single Cell Experiment and Scanpy: Most of the R packages are available from CRAN and can be installed and accessed as mentioned below:
    install.packages('Seurat')
    library(Seurat)
    This will install the updated version of the softwares.
    Once you download the expression matrix and metadata from HCA (Human Cell Atlas), process the data to obtain the h5ad file format (a gene expression matrix file format) as stated below in R version 4.0.2.
    library(SingleCellExperiment)
    library(scran)
    library(sceasy)
    library(reticulate)
    #SET working directory pointing to the folder where all your
    files are to be saved and the outputs will be saved at that
    location
    setwd(/PATH/TO/YOUR/DIRECTORY)
    # Read matrix
    airway.data <- read.table(file="exprMatrix_hg19.tsv", header =
    T, row.names=1, sep="\t", as.is=T)
    #Normalize
    airway.data_log <- log(airway.data + 1)
    airway.data_log_mtx <- data.matrix(airway.data_log)
    write.csv(airway.data_log_mtx, file =
    exprMatrix_norm_hg19.tsv")
    #Identify Highly variable genes
    mgv <- modelGeneVar(airway.data_log_mtx)
    top_hvg <- getTopHVGs(mgv)
    my_metadata <-
    >read.csv("meta_cell_types_merged.tsv",sep="\t")
    #generate SCE object
    sce <- SingleCellExperiment(assays = list(logcounts = airway.data_log),colData = my_metadata)
    use_condaenv('EnvironmentName')
    loompy <- reticulate::import('loompy')
    sceasy::convertFormat(sce, from="sce",
    to="anndata",outFile='deprez_cell_types_merged.h5ad')
    Analyze the h5ad output file generated from the above step using Scanpy library (in Python). Perform principal component analysis (PCAs), select the highly variable PCAs for cluster detection (PhenoGraph) and calculate the cluster connectivity using the partition-based graph abstraction (PAGA) algorithm. The python codes are described in the GitHub repository at Github data: https://github.com/theislab/scanpy. The control dataset 2 can be downloaded as h5ad object and similar steps for filtering, normalization, PCA calculation and clustering can be done using Scanpy. Use UMAP (uniform manifold approximation and projection) to visualize the clusters in reduced dimensional space, and then compare the cluster topography with the UMAP from the original studies. Identify the number of PCAs using elbow plot for data clustering and decide on the highly variable features to be used for downstream analysis.
  b. COVID single cell dataset processing using Seurat: Once you download the data from GSE145926, process it using Seurat in R, Github data: https://github.com/zhangzlab/covid_balf/ seurat_integration.R). The script ‘seurat_integration.R’ carries out the following functions.
    i. Load data and create Seurat object.
    ii. Preprocess data, filtering the matrix with nFeature_RNA, nCount_RNA, and percent.mito.
    iii. Integrate data using Seurat v3.
    iv. Scale data in ‘Integrated’ assay and compute PCAs.
    v. Select the number of PCs using elbow plot and heatmap.
    vi. Cluster the cells and visualize using UMAP.
    vii. Scale data in ‘RNA’ assay.
    viii. Find markers for every cluster and visualize their expression using heatmap/feature plot/violin plot/ dot plot.
      If using R-studio, copy and paste the code from GitHub in a new R-Script. Select the whole script and press the ‘RUN’ icon and observe your outputs in the console window. Else, you can save this code as ‘seurat_integration.R’, open R console on terminal by writing R, change and set the directory and execute seurat_integration.R as shown below. Quit the screen after running the code successfully.
      %R
      #R version will be printed on your screen
      setwd("∼/PATH_TO_WORKING_DIRECTORY_WHERE_db.R_IS_SAVED")
      seurat_integration.R
      q()
      Compare the UMAP with the one generated by (Liao et al., 2020).
    ix. Subset the Seurat object using group identity details from the metadata. In this case, we have three groups: Severe(S), Control (C) and Moderate (M) COVID-19 patient data.
    x. Save each one as a separate R object (as shown below) for further processing as per standard steps (Deprez et al., 2020; Liao et al., 2020; Madissoon et al., 2019; Nassir et al., 2021a; Tambi et al., 2021).
      severe<-subset(Seurat_object, idents= “S”)
      control<-subset(Seurat_object, idents= “C”)
      moderate<-subset(Seurat_object, idents= “M”)
      saveRDS(severe, file=”/PATH_TO_FILE/severe.rds”)
      saveRDS(moderate, file=”/PATH_TO_FILE/moderate.rds”)
      saveRDS(control, file=”/PATH_TO_FILE/control.rds”)

2. Prepare signature gene marker dataset and ascertain cell type identity of clusters.
  This section describes steps in marker dataset preparation and assigning cell type identity to clusters.
  a. Dataset preparation.
    Marker dataset compilation will depend on the type of single cell data you are processing. Here, we composed an in-house database consisting of canonical markers for cell types associated to human lung region using a combination of literature search (Deprez et al., 2020; Grant et al., 2021; Madissoon et al., 2019; Reyfman et al., 2019), PanglaoDB (Franzen et al., 2019), and CellMarker (Zhang et al., 2019) databases. Deprez et al. provided the single cell map for human healthy airway, constituted by 77,969 cells from 35 different locations, which were clustered into seventeen broad cell types. We extracted the unique markers from the heat map representing the marker gene expression across various cell types. Furthermore, we collected the marker genes from lung single cell map (Madissoon et al., 2019) consisting of 57,020 cells, classified into 25 different cell types. Similarly, we enlisted the lung specific markers from other largescale datasets, which characterized single cell heterogeneity of lung tissues. The steps for compiling marker genes are shown in Figure 1.
    In order to download the marker gene files from PanglaoDB and CellMarker database, first install curl on your PC through terminal window executing the following codes.
    #install curl
    sudo apt install curl
    #check if curl is installed
    curl --version
    Next, open either R-studio or R-terminal and execute the following code which will:
    i. Download the data from PanglaoDB and CellMarker database (You can either download the file or use the file we have deposited in Zenodo).
    ii. Select human, lung data which have sensitivity above 0.5 and keep the genes associated with each cell type in a new list.
    iii. Identify only unique list of genes for each cell type and combine all the cell types (collected from literature as well as databases - Figure 1) to generate the final database. In our study, we compiled 966 unique marker genes representing 38 cell types (Nassir et al., 2021b).
      library(curl)
      library(dplyr)
      #PanglaoDB
      url <-
      "https://panglaodb.se/markers/PanglaoDB_markers_27_Mar_2020.tsv.gz"
      dest<- "/Users/ Desktop/STAR_protocol-
      _review_comments/new/PanglaoDB_markers_27_Mar_2020.tsv.gz"
      curl_download(url,dest)
      #tsv=gzfile("PanglaoDB_markers_27_Mar_2020.tsv.gz")
      #or
      tsv=("PanglaoDB_markers_27_Mar_2020.tsv")
      pdb<- read.csv(tsv,header=T,sep = "\t")
      pdb_filtered<-filter(pdb, species == "Hs" | species == "Mm Hs")
      pdb_filtered<-filter(pdb_filtered,organ=="Lungs")
      pdb_filtered<-filter(pdb_filtered,sensitivity_human>=0.5)
      pdb1 <- split(as.character(pdb_filtered$official.gene.symbol), pdb_filtered$cell.type)
      View(pdb1)
      #Cellmarker
      url2<-
      "http://biocc.hrbmu.edu.cn/CellMarker/download/Human_cell_markers.txt"
      dest2<- "/Users/ Desktop/STAR_protocol-
      _review_comments/new/Human_cell_markers.txt"
      curl_download(url2,dest2)
      cmdb<- read.csv("Human_cell_markers.txt", header=T, sep="\t")
      cmdb_filtered<-filter(cmdb,cancerType=="Normal")
      cmdb_filtered<-filter(cmdb_filtered,tissueType=="Lung")
      cmdb1<-split(as.character(cmdb_filtered$geneSymbol),cmdb_filtered$cellName)
      View(cmdb1)
      # cmdb1[["T-cell"]]<-c("CD3D", "CD3E", "CD3G", "CD4", "CD8A","CD4",
      "CD68", "CD8A")
      lis<-names(pdb1)
      lis2<-names(cmdb1)
      #making list of all cell types
      nam_all_cell<-unique(unlist(c(lis,lis2)))
      #compare cell type names
      #merge
      #Compare genes within- keep only unique
      for(j in 1:length(nam_all_cell)){
       d=e=g=h=c()
       if(j==8)
       {
       g<-grep("Brush cell",lis2) #Brush cell labeled as Brush cell (Tuft
      cell) –it gives error using Grep
       }
       else if(j==18){
       g<-grep("FOXN4",lis2) #FOXN4 labeled as FOXN4 +) –it gives error
      using Grep due to ‘+’
       }
       else{
       g<-grep(nam_all_cell[j],lis2)
       }
       d<-length(g)
       h<-grep(nam_all_cell[j],lis)
       e<-length(h)
      if((d>0)&(e>0)){
       x<-unique(unlist(pdb1[h]))
       y<-unique(unlist(cmdb1[g]))
       ip<- unique(unlist(c(x,y)))
       nam_ip<-names(cmdb1[g])
       }else if(d>0){
       ip<-unique(unlist(cmdb1[g]))
       nam_ip<-names(cmdb1[g])
       }else if(e>0){
       ip<-unique(unlist(pdb1[h]))
       nam_ip<-names(pdb1[h])
       }
       DF<-as.data.frame(ip,col.names=nam_ip,row.names=NULL)
       DF<-t(DF)
       row.names(DF)<-nam_ip
       write.table(DF,file="cell_Type.csv",row.names =
      TRUE,col.names=FALSE, append=TRUE, sep=",")
       print(DF)
       }
      If you are using R-studio, copy and paste the above code in the new R-Script. Select the whole script and press the ‘RUN’ icon and observe your outputs in the console window. Else, you can save this code as ‘db.R’, open R console on terminal by writing R, change and set the directory and execute db.R as shown below. Quit the screen after running the code successfully.
      %R
      #R version will be printed on your screen
      >setwd("∼/PATH_TO_WORKING_DIRECTORY_WHERE_db.R_IS_SAVED")
      >db.R
      >q()
      **CRITICAL:** After collecting data from each source, it is critical to carefully check the cell type names. Even minor difference in names (cell vs Cell vs CELLS) might lead to creation of redundant cell types (ex. Brush cell vs Brush cells - both are the same and should be combined into one).
      **CRITICAL:** It is critical to exhaustively search the publications for marker database creation as the quality of this database will be reflected directly on cell type assignment. Only going through the databases will hardly take few minutes to hours. It is the literature search which is time consuming, due to the exponential rate at which single cell analysis is being performed on different human tissues.
  b. Cell type assignment
    Subsequently, use the marker dataset to find the cluster identity (Figure 2) employing these steps:
    i. Extract and plot (boxplot) the normalized expression for all the genes belonging to a specific cell type across all the clusters for the given dataset. Alternatively, you can also calculate the median expression for all the clusters per cell type in the database. The following code can be used for calculating the median.
      library(readxl)
      library(Seurat)
      library(SeuratData)
      library(SeuratDisk)
      library(ggplot2)
      library(glue)
      my_data <- read_excel("cell_type_db1.xls")
      severe<-readRDS("/Volumes/TR/8thDec2021/Download/severe.rds")
      len<-length(my_data)
      nl<-c()
      feat_new<-c()
       for(i in 1:len){
       nl<-c()
       k=1
       feat<-my_data[[i]]
       u<-length(feat)
       for(j in 1:length(feat)){
       if(is.na(feat[j])){
       k=0
       j=(length(feat))+1
       }
       else{
       nl[[k]]<-feat[[j]]
       k=k+1
       }
       }
      g<-glue_collapse(nl,sep=" ")
       feat_new[i]<-g
       io<-unlist(strsplit(feat_new[i], " "))
       print(io)
       print(length(io))
      }
      length(feat_new)
      t<-c()
      for(q in 1:38){
      io<-unlist(strsplit(feat_new[q], " "))
      print(io)
      #change cluster number and calculate for each cluster
      sev <- as.matrix(FetchData(object=severe, vars = io,
      cells=c(names(severe$seurat_clusters[severe$seurat_clusters==10]))))
      values<-as.list(sev)
      h<-dim(sev)[1]∗dim(sev)[2]
      length(values)
      val75<-round((0.90∗length(values)),digits=0)
      sev1<-sev[order(sev)]
      sev11<-sev1[val75:length(sev1)]
      df<-data.frame(expr=sev11)
      t[q]<-median(df$expr)
      }
      #ranking the median values
      hig_od<-order(t,decreasing=T)
      median_desc<-t[order(t,decreasing=T)]
      top<-hig_od[1]
      paste("The max median value is for ", colnames(my_data)[top], "of",
      median_desc[1], "for cluster 1" )
      #If u need box plot
      write.csv(df,"sev.csv", row.names = FALSE)
      #Add column headin as 'Sam' for sample and 'Exp' for expression values
      and mention cluster number for the 1st row
      #Sam Exp
      #Cluster1 0
      #Cluster1 7.092988176
      #Cluster1 6.943530007
      expr <- read.csv("sev.csv")
      g <- ggplot(expr, aes(x=Sam, y=Exp, fill=Sam))
      g + geom_boxplot()+geom_point()+ggtitle("Cell_type_under_investigation")
    ii. Compare the median expression across the cell types per cluster and to the 99th percentile expression value from the datasets.
      library(Seurat)
      severe<-readRDS("/PATH/TO/SEURAT_R_DATA_FILE/ severe.rds")
      all.genes <- rownames(Your_seurat_object)
      rt<-FetchData(Your_seurat_object, vars<-all.genes)
      q99<-quantile(rt,0.99)
    iii. Assign the cell type identity based on the highest normalized median expression value for each cluster.

3. Prepare comorbid gene list and identify subtype with upregulated comorbid gene expression.

This section describes steps in compiling the list of COVID-19 comorbid gene lists and identifying the cluster with maximum number of enriched comorbid gene set.

We collated a list of COVID-19 associated genes to analyze and compare their expression among control, moderate and severe COVID-19 clusters. Since COVID-19 disease pathology is associated with enormous release of pro-inflammatory cytokines, we included the list of cytokines and cytokine receptor genes, in addition to the rare infection and syndromic genes accounting for the genetic predisposition, lung channelopathy genes, differentially expressed genes from COVID-19’s BALF transcriptome (Zhou et al., 2020) and genes associated with key comorbid conditions such as chronic obstructive pulmonary disorder, cardiovascular disease, hypertension and diabetes. The list of cytokine and cytokine-receptor genes was retrieved from the Immunology Database and Analysis Portal (Bhattacharya et al., 2018). The monogenic infection gene list was collected from (Casanova, 2015). Syndromic genes (category S) were collected from the Simons Foundation Autism Research Initiative (https://gene.sfari.org/). Primary comorbid disease genes associated with COVID-19 were extracted from GWAS catalog (https://www.ebi.ac.uk/gwas/), where the selected genes had a probability < 10^-7^. The normalized expression of these gene sets was plotted for all the clusters of control, moderate and severe datasets. Refer the script at Github data: https://github.com/MBRULab/2021_Nassir-etal/blob/main/comorbidexpression.R. If using R-studio, copy and paste the code from GitHub in new R-Script. Select the whole script and press the ‘RUN’ icon and observe your outputs in the console window. Else, you can save this code as ‘comorbidexpression.R’, open R console on terminal by writing R, change and set directory and execute comorbidexpression.R as shown below. Quit the screen after running the code successfully.

The script below will yield cluster specific boxplots for comorbid gene expression.

Mark clusters for which the normalized median expression of the comorbid condition associated gene sets was higher than 99th percentile expression value from the dataset. Compare the boxplots and choose the cluster in which most of the comorbid conditions is upregulated as your subtype of interest, as demonstrated in Figure 2 and Figure S5 of (Nassir et al., 2021b).

%R

#R version will be printed on your screen

>setwd("∼/PATH_TO_WORKING_DIRECTORY_WHERE_db.R_IS_SAVED")

> comorbidexpression.R

>q()

**Figure 3:**
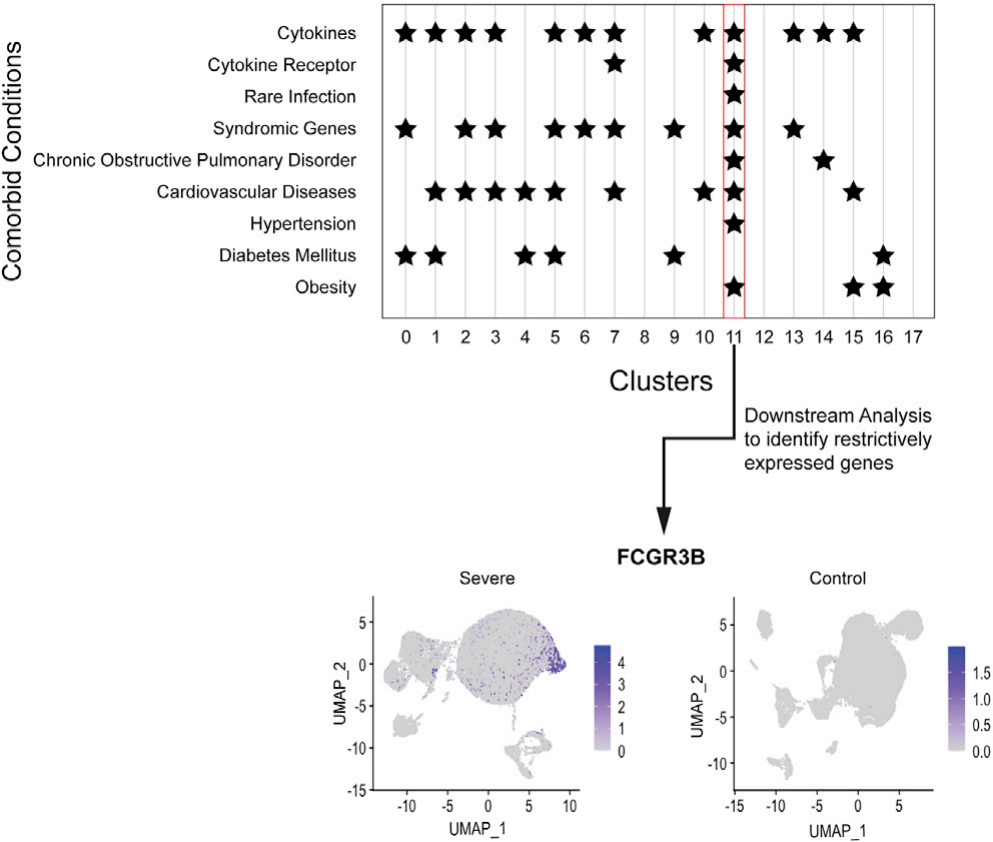
Steps in identifying cluster associated with comorbid disease gene expression and finding gene restricted to that cell type Enrichment analysis of severe COVID-19 clusters with comorbid gene set. Higher expression (above global median) is indicated by a star. The cluster having maximum number of upregulated gene set is selected for further downstream analysis and identifying candidate genes with restrictive expression in severe COVID-19 cluster. The representative feature plots are reused from Figure 3 of (Nassir et al., 2021b).

4. Downstream analysis.
  This section describes identifying genes restricted to cell type of interest.
  Using step 3, we identified a severe cluster which has maximum enrichment of the comorbid genes, severe cluster 11, which was assigned the identity as monocyte-derived alveolar macrophages (MoAM), indicated by the presence of *CCL3L1 -* MoAM_CCL3L1. One of the most important steps next is to identify the marker genes that are restrictively expressed in this cluster. Please follow the steps below.
  a. Use FindAllMarkers to compute the DEGs for each cluster using different tests - Wilcoxon-ranked sum test, t-test.
    >FindAllMarkers(seu_object, test.use= “wilkox”)
  b. Select the genes which are differentially expressed (highest probability, combining all the three tests).
  c. Rank the genes using their Log of fold change in descending order.
  d. Use top 20 genes and map them using feature plot and dot plot. Analyze the expression pattern and select the genes which are upregulated in severe cluster and have negligible expression in all other clusters in the dataset.
    Using this approach, we identified the most differentially regulated gene that demarcated cluster 11 in samples from severe COVID-19 patients, *FCGR3B* (Figure 3).

5. Pathway enrichment.

This section describes pathway enrichment of differentially expressed genes and visualization of pathways using Cytoscape tool.

**Figure 4:**
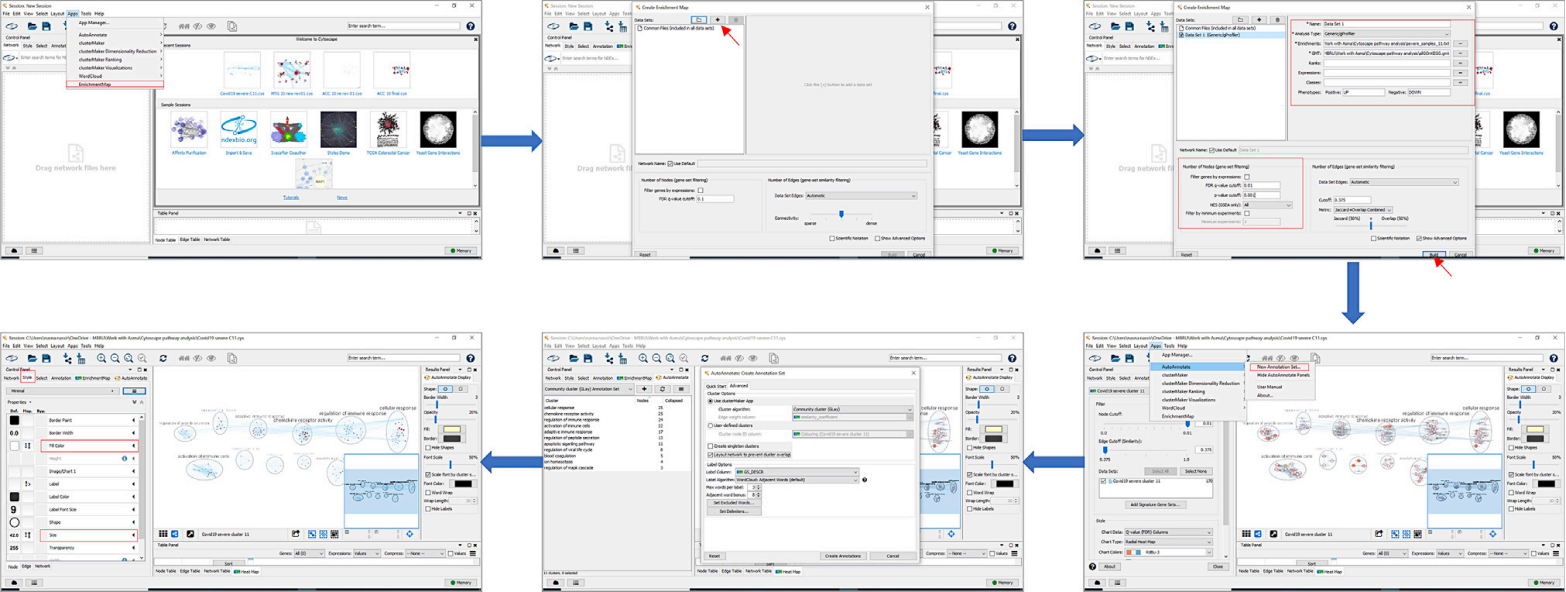
Steps to perform Cytoscape analysis Flowchart representing pathway network creation using Enrichment Map and Autoannotate tool. The tab selections are highlighted in red.

We used the DEGs from severe cluster 11 for enrichment analysis and mapping. For enrichment analysis, please follow the script at Github data: https://github.com/MBRULab/2021_Nassir-etal/blob/main/geneoverlap.R. This script takes the list of genes from severe cluster 11 as input and scans the KEGG (Kyoto Encyclopedia of Genes and Genomes) and GO (Gene Ontology) databases to identify the overlapping pathways. The input file required (KEGG and GO file and gene list file) are deposited in Zenodo. Here, the intersection of your set of genes over KEGG and GO pathway genes is computed. This script generates a csv file with the following information: pathway id, pathway name, pvalue and Odds ratio. This file is used as the input to Cytoscape where two main plugins are used: ‘Enrichment map’ and ‘Autoannotate’. Cytoscape is an open-source network visualization software which can be downloaded from https://cytoscape.org/download.html. Further, ‘App Manager’ present in the ‘APPS’ tab within the Cytoscape can be used to add these plugins (Figure 4). Then, use the Enrichment map and input the above-mentioned files using ‘Generic/gprofiler’ type of analysis. Set the FDR cut off (<0.01) OR p value cut off (<0.001) (after clicking the advanced icon). After building the map, use the auto annotate plugin and refine the network map.

**CRITICAL:** This script restricts the pathway to size 50 to 1,000 genes (which means only pathways having more than 50 and less than 1,000 genes are included – to account for housekeeping genes), that can be modified on line 17 of the script.

**CRITICAL:** We consider a pathway intersection > 5 as positive match. It means that pathway will be considered only if more than 5 genes are included in it. This can be relaxed (by reducing the number) on line 26 of the script.

6. In silico validation.

This section describes validation of findings using other single cell and bulk RNA-seq datasets.

Once the cell type and specific marker gene has been identified, you will need to validate your findings, for which you need to decide and download required validation data sets. We used single cell BALF, bulk PBMC and bulk nasopharyngeal datasets for validation. This step involved comparing the expression pattern of *FCGR3B* in control and patient data and then calculating the significance using t-test p-values. The validation script deposited at Github data: https://github.com/MBRULab/2021_Nassir-etal (validation_BALF_SC.R, validation_nasph.R, validation_pbmc.R) can be directly used for this purpose. . If using R-studio, copy and paste the code from GitHub in new R-Script. Select the whole script and press the ‘RUN’ icon and observe your outputs in the console window. Else, you can save this code as ‘validation.R’, open R console on terminal by writing R, change and set directory and execute validation.R as shown below. Quit the screen after running the code successfully.

%R

#R version will be printed on your screen

>setwd("∼/PATH_TO_WORKING_DIRECTORY_WHERE_db.R_IS_SAVED")

> validation.R

>q()

7. Gene expression in severe COVID-19 clinical samples.
  This section describes processing of nasopharyngeal swabs for RNA isolation followed by real time PCR analysis.
  a. Transfer nasopharyngeal swabs into viral transport media utilizing BD Universal Transport Media (UTM) 3-mL collection kit with flexible minitip flocked swab (BD, Cat. No. 220531).
  b. Perform RT-qPCR for SARS-CoV-2 using the QIAamp Viral RNA Mini or the EZ1 DSP Virus Kits (QIAGEN, Hilden, Germany) following the manufacturer’s recommended protocol.
  c. Extract human RNA from nasopharyngeal swabs of control (SARS-CoV-2 negative) and severe/critical cases (pneumonia requiring admission to intensive care units and specialized life-support treatment (e.g., mechanical ventilation)) using RNeasy 96 QIAcube HT Kit (QIAGEN USA) as per the manufacturer’s instructions.
    **CRITICAL:** Pay attention to avoid RNase contamination.
  d. Transfer 2 mL of Universal Transfer Medium (UTM) to a clean, sterile tube and centrifuge at 1,800 g for 3 min. Carefully remove 1,650 μL of supernatant without disturbing the pelleted squamous and respiratory epithelial cells.
  e. Resuspend the pellet with remaining 350 μL of Universal Transfer Medium (UTM) and transfer to S Block (RNeasy 96 QIAcube HT Kit, QIAGEN USA).
  f. Determine quality and quantity of the extracted RNA using NanoDrop™ 8000 Spectrophotometer (Thermo Fisher Scientific, USA).
  g. Reverse transcribe RNA to cDNA using a High-Capacity cDNA Kit (Applied Biosystems, Foster City, CA) according to the manufacturer’s protocol. Prepare master mix for reverse transcriptase reaction in a microcentrifuge tube.
    RT Master Mix for cDNA Generation

**Table:** 

Reagent	Volume per reaction (μL)
Multiscribe reverse transcriptase	1
25× dNTP Mix	0.8
10× RT Random Primers	2
RNase inhibitor	1
10× RT Buffer	2
Nuclease free water	3.2
Total Volume	10
  h. Mix the reagents by briefly pipetting or gently vortexing. Label tubes and pipette 10 μL of master mix into PCR strip tubes. Add 10 μL RNA to respective PCR strip tube and gently mix by pipetting 5–10 times.
  i. Flash spin PCR strip tubes in a mini centrifuge at 2,000 g for approximately 10 s. Place tubes into thermocycler and run the following program:
    cDNA generation PCR steps

**Table:** 

Steps	Temperature	Time
1	25°C	10 min
2	37°C	120 min
3	85°C	5 min
4	4°C	Infinity
  j. Thaw TaqMan Fast Advanced Master Mix on ice. Thaw primer/probe set on ice, protected from light. Label one microcentrifuge tube for each primer/probe set and mix the master mix and primer/probes.
    Two-Step Multiplex qPCR Master Mix Recipe

**Table:** 

Reagent	Volume per reaction (μL)
2× TaqMan Fast Advanced Master Mix	5
20× TaqMan probe1	0.5
20× TaqMan probe2	0.5
Nuclease-free H2O	3
Final total volume	9

    ***Note:*** Use technical duplicates for each sample and primer/probe set.
  k. Prepare the above mixture and mix by pipetting slowly up and down. Dispense 9 μL of master mix reaction into a 96-well plate. Add 1 μL cDNA to the 96-well plate containing the reaction mixture. Cover the loaded 96-well plate with an adhesive cover and centrifuge the 96-well plate in a mini plate spinner at 500 g for approximately 20 s. Select the reagent type as TaqMan reagents. Choose the ramp speed and plate setup. Define the specific probe and samples, fluorescence reporter and the quencher for the experiment. Assign the probes and samples to selected wells.
    **CRITICAL:** Include a no-template control. Prior to initializing the run, assign target genes with the appropriate fluorophore (GAPDH-VIC, FFAR2-FAM).
  l. Set up the following program:
    PCR cycling condition

**Table:** 

Steps	Temperature	Time	Cycles
Initial incubation	50°C	2 min	1
Polymerase activation	95°C	5 min	1
Denaturation	95°C	1 s	40 cycles
Annealing	60°C	20 s	40 cycles
Hold	4°C	Forever	

Place the 96-well plate in the machine and start the Run method. Edit the default run method with appropriate Tm. Include Pre-PCR read to collect the background fluorescence.
  m. Data Analysis - Review the QC summary (select amplification plot to show amplification curves in all wells. Specific PCR products should display a single sharp peak in the melting curve rather than multiple peaks or single broader peaks (non-specific PCR product)).
  n. Calculate relative levels of mRNA gene expression using the 2^−ΔΔCT^ method and plot fold change.
    Relative gene expression = 2^−ΔΔCT^, where ΔCT is the difference in CT value of target gene with respect to housekeeping gene, ΔΔCT is the difference in CT value of target gene of patients with respect to control.
8. Gene expression in spike protein stimulated obese subjects.
  This section describes culturing lung epithelial cells, monocyte isolation and culturing followed by real time PCR analysis.
  a. Lung epithelial cell culture.
    i. Purchase Normal human primary bronchial epithelial (NHBE) cells from non-obese and obese subjects from a commercial source (key resources table).
    ii. Culture NHBE cells in BEGM media (Lonza, MD, USA) supplemented with 1% antibiotic antimycotic solution (Wisent, QC, CA) in 12 well tissue culture plates coated with Type 1 Rat tail collagen (Sigma-Aldrich, Ontario, Canada).
    iii. Grow cells to 90% confluency and starve using BEBM Basal Medium (Lonza, MD, USA) supplemented with 1% antibiotic antimycotic solution (Wisent, QC, CA) over night.
    iv. The next day, stimulate cells with 1 μg/mL of SARS-CoV-2 spike protein (S1+S2) for 3 h.
    v. Collect the culture media in microtubes, centrifuge at 5,000 g for 5 min. Aliquot supernatants into fresh microtubes and freeze at −80°C.
  b. Monocyte isolation and culture.
    i. Isolate PBMCs of a healthy donor from 40 mL of blood using SepMate-50 tubes (StemCell, BC, CA), following manufacture’s protocol.
    ii. Resuspend PBMCs in RPMI 1640 supplemented with 1% Penicillin/Streptomycin, Glutamax and 10% FBS (Wisent, QC, CA) (Gibco, MD, USA) (Wisent, QC, CA).
    iii. Perform differential cell count on the PBMC cell suspension using the Beckman Coulter AC-T DIFF cell counter and obtain concentration of monocytes.
    iv. Add calculated volume of PBMC cell suspension to a 48 well plate to obtain 1 × 10ˆ5 monocytes per well.
    v. Incubate cell suspension at 37°C, 5% CO2 for 3 h. Remove media from wells and wash twice with PBS.
    vi. Add fresh complete RPMI media with 0.5% FBS to each well and incubate attached monocytes (16 h). The following day, thaw an aliquot of conditioned media from stimulated NHBE cells on ice and vortex.
    vii. Remove 100 μL of media from wells and add 100 μL of NHBE conditioned media to attached monocytes and incubate for 24 h. Remove media from cells and use cell pellets for RNA extraction.
    viii. Perform total RNA extraction from monocytes using phenol-chloroform extraction (RiboZol RNA extraction reagent, VWR, Leicestershire, UK), as directed in the manufacturer’s instructions.
    ix. Remove contaminated DNA from 500 ng of total RNA using the AccuRT Genomic DNA Removal Kit (Applied Biological Materials, Richmond, BC, Canada), following the manufacturer’s protocol.
      DNA Removal for cDNA Generation

**Table:** 

Reagent	Volume per reaction (μL)
RNA (500 ng)	up to 6
AccuRT Reaction mix	2
Nuclease free water	up to 6
Incubate 5 min	
Reaction Stopper	2
Total Volume	10
    x. Perform reverse transcription using the 5× All-In-One Reverse Transcriptase Mastermix (ABM).
      RT Master Mix for cDNA Generation

**Table:** 

Reagent	Volume per reaction (μL)
5× All-in-One RT master mix	4
Nuclease free water	6
Total Volume	20
      cDNA generation PCR steps

**Table:** 

Steps	Temperature	Time
1	25°C	10 min
2	42°C	15 min
3	85°C	5 min
4	4°C	Infinity
    xi. Measure mRNA expression of *FCGR3B* and *GAPDH* (housekeeping gene) using EvaGreen qPCR Mastermix (ABM).
    xii. Test each sample in duplicates and perform qPCR amplification using CFX96 thermal cycler (Bio-Rad, Hercules, 130 CA, USA).
      qPCR Master Mix Recipe

**Table:** 

Reagent	Volume per Reaction (μL)
2× EvaGreen Mastermix	5
10 μM Forward & Reverse primers	0.6
Nuclease free water	2.4
Total Volume	10
    xiii. Set the cycler program:
      PCR cycling condition

**Table:** 

Steps	Temperature	Time	Cycles
Polymerase activation	95°C	20 s	1
Denaturation	95°C	3 s	40 cycles
Annealing	60°C	30 s	40 cycles
Hold	4°C	Forever
    xiv. Use the ΔCT method to measure gene expression: amount of target = CT ref /ΔCT.

## Expected outcomes

Using this protocol, it is possible to process scRNA-seq data to identify cell types enriched for any gene set and determine markers associated with disease/pathway. The protocol will guide you to perform single cell clustering and data analysis, pathway analysis, real time PCR and cell culture.

## Limitations

Potential confounders that can affect the outcome of this kind of study are lack of sample replicates and the batch effect arising from different samples. Batch effect is technical source of variation which affects the data quality and the subsequent downstream analysis (Tran et al., 2020). Here, our analysis was conducted independently using batch corrected data for each single cell (SC) transcriptome dataset without combining them. Hence, our analysis did not have any mix of batch affect. However, while using data from different sources and integrating them, batch effect should be corrected. The inclusion of sample replicates is subjected to availability of patient samples and cost incurred in single cell RNA sequencing. The small sample size of this cohort is a limitation (31 severe COVID-19 patients and 11 healthy controls). Although we did qPCR validation for *FCGR3B* using nasopharyngeal swabs (due to availability), the ideal validation sample would be flow cytometry sorted macrophages from BALF region to match the observed upregulated *FCGR3B* expression in MoAM of BALF region using single-cell RNA-seq data *in silico*.

## Troubleshooting

### Problem 1

Unable to reproduce the UMAP which matches the original work.

### Potential solution

In the preprocessing steps, the single cell transcriptome data will be available in different formats (Seurat, h5ad, loom and so on). It is important to use the same steps and the single cell analysis tool as used by original work and then converting the final output to the desired format for further processing. The overall topology of the UMAP will be preserved for comparison purpose.

### Problem 2

Assigning of the cell type identity will be affected if the marker dataset is limited and consists only of a few canonical markers. As discussed in Figure S2 (Nassir et al., 2021b), limited markers will lead to vague cell type identification, for example – many clusters might represent same cell types, viz. macrophage representing many clusters.

### Potential solution

It is always better to refer multiple resources (Human cell atlas, databases like CellMarker and PanglaoDB and latest peer reviewed research articles). A comprehensive marker database will aid in unbiased cell type mapping. Each gene should be present only once defining a specific cell type. In case of conflicting genes/cell types, that gene/cell type should be removed to maintain a unique cell type marker database.

### Problem 3

Low RNA yield from nasopharyngeal swabs.

### Potential solution

Some of the swab Universal Transfer Medium (UTM) may not contain enough number of cells, so, if the RNA yield is less, this step can be repeated until we get minimum number of cells accumulated to extract required amount of RNA.

### Problem 4

Non-standard gene names will lead to improper downstream analysis.

### Potential solution

It’s better to retrieve gene names from NCBI/Gene Cards. Care should be taken especially while retrieving from literature. For example, the same gene can be mentioned as ILR2 and IL2RA. Such conflicts should be carefully checked and resolved by comparing with NCBI nomenclature.

***Alternatives:*** 1. Different clustering tools, cell type databases, parameters or statistical tests can be used for analyzing single-cell RNA-seq data. 2. The severe, moderate and control data can be either clustered separately or merged depending on the objective of the study. 3. Automated cell type identification tools can be used instead of the cell type annotation described in the protocol.

## Resource availability

### Lead contact

Further information and requests for resources and reagents should be directed to and will be fulfilled by the lead contact, [Dr. Mohammed Uddin, Email: mohammed.uddin@mbru.ac.ae].

### Materials availability

This study did not generate new unique reagents.

## Data Availability

Complete datasets used for this analysis are deposited in Zenodo data: https://doi.org/10.5281/zenodo.5809990. Analyses were conducted in R and Python; the accession number for all codes reported in this paper have been deposited as Github data: https://github.com/MBRULab/2021_Nassir-etal. Any additional information required to reanalyze the data reported in this paper is available through personnel contact with the corresponding author upon request.
